# Cellular Receptors Involved in KSHV Infection

**DOI:** 10.3390/v13010118

**Published:** 2021-01-17

**Authors:** Emma van der Meulen, Meg Anderton, Melissa J. Blumenthal, Georgia Schäfer

**Affiliations:** 1 International Centre for Genetic Engineering and Biotechnology (ICGEB), Observatory, Cape Town 7925, South Africa; vmlemm001@myuct.ac.za (E.v.d.M.); andmeg005@myuct.ac.za (M.A.); 2 Institute of Infectious Disease and Molecular Medicine (IDM), Faculty of Health Sciences, University of Cape Town, Observatory, Cape Town 7925, South Africa; 3 Division of Medical Biochemistry and Structural Biology, Department of Integrative Biomedical Sciences, Faculty of Health Sciences, University of Cape Town, Observatory, Cape Town 7925, South Africa

**Keywords:** Kaposi’s Sarcoma Herpes Virus (KSHV), Kaposi’s sarcoma, Eph receptors, endothelial cells

## Abstract

The process of Kaposi’s Sarcoma Herpes Virus’ (KSHV) entry into target cells is complex and engages several viral glycoproteins which bind to a large range of host cell surface molecules. Receptors for KSHV include heparan sulphate proteoglycans (HSPGs), several integrins and Eph receptors, cystine/glutamate antiporter (xCT) and Dendritic Cell-Specific Intercellular adhesion molecule-3-grabbing non-integrin (DC-SIGN). This diverse range of potential binding and entry sites allows KSHV to have a broad cell tropism, and entry into specific cells is dependent on the available receptor repertoire. Several molecules involved in KSHV entry have been well characterized, particularly those postulated to be associated with KSHV-associated pathologies such as Kaposi’s Sarcoma (KS). In this review, KSHV infection of specific cell types pertinent to its pathogenesis will be comprehensively summarized with a focus on the specific cell surface binding and entry receptors KSHV exploits to gain access to a variety of cell types. Gaps in the current literature regarding understanding interactions between KSHV glycoproteins and cellular receptors in virus infection are identified which will lead to the development of virus infection intervention strategies.

## 1. Introduction

Kaposi’s sarcoma-associated herpesvirus (KSHV), or human herpesvirus-8 (HHV-8), is an oncogenic γ-herpesvirus [1] which is the etiological agent of the most prevalent AIDS-related malignancy, Kaposi’s sarcoma (KS) [2]. KSHV is also the causative agent of two lymphoproliferative disorders, the rare Multicentric Castleman disease (MCD) and primary effusion lymphoma (PEL). KSHV inflammatory cytokine syndrome (KICS) can also be attributed to KSHV infection [3,4,5,6,7].

Saliva is thought to be the primary route of transmission, however KSHV has also been detected in breast milk, semen and blood [8,9]. As is typical for herpesviruses, KSHV has a very broad host cell tropism in vitro and in vivo. In vivo, KSHV can infect endothelial cells, B cells, epithelial cells and fibroblasts, to name a few significant examples [10]. Herpesviruses typically engage multiple cell surface receptors with their envelope glycoproteins to gain access. Some of these host molecules are required for binding to concentrate the virus on the cell surface, while others facilitate entry [11]. The cellular receptor(s) that KSHV binds to is dependent on its abundance or expression level on a particular cell, allowing KSHV its broad host cell tropism.

Following binding and making use of cellular signaling molecules, KSHV enters cells utilizing diverse endocytic pathways including clathrin- and caveolin-mediated endocytosis, macropinocytosis and undefined endocytic entry pathways, depending on the cellular context [12]. Thereafter, the viral envelope fuses with the membrane of the endosome, likely triggered by low pH as in other herpesviruses, and the capsid is released into the perinuclear region. The KSHV genome enters the nucleus via nuclear pores where the linear genome rapidly undergoes circularization into an episome [13,14,15,16].

This review comprehensively summarizes the current knowledge on cellular receptors involved in KSHV infection of diverse cell types implicated in its pathogenesis to supplement recent reviews covering viral glycoproteins involved in KSHV infection [16] and infection in the lymphocyte compartment [17].

## 2. KSHV Envelope Glycoproteins

KSHV is an enveloped, double-stranded DNA virus with a genome of 165 kb [18,19]. Preliminary attachment to the host cell surface and the subsequent entry of KSHV is mediated by glycoproteins embedded in the virus envelope. The virion envelope contains several conserved herpesvirus glycoproteins, namely gB, gH, gL, gM and gN, which are encoded by the Open Reading Frames (ORFs) 8, 22, 47, 39, and 53, respectively [19,20,21]. The glycoproteins gpK8.1A and B, ORF4, ORF27, ORF28 and ORF68, associated with the lytic cycle, and ORF45, an RSK activator protein, are unique to KSHV [19,21,22,23,24,25,26,27,28]. The glycoproteins considered essential to KSHV entry are K8.1, gB and the gH-gL heterodimer, and their engagement with specific cellular receptors is covered in Section 3. The particular repertoire of cellular receptors available to engage with specific glycoproteins eventually leads to a concerted series of molecular events culminating in fusion of the viral envelope with the host cell membrane. It is widely accepted that gB, which is comprised of five functional domains typical for type III fusion glycoproteins, is the initial cell binding protein [29] and key fusogen leading to virus entry and infection, and that low pH may facilitate gB-mediated KSHV fusion [16]. A study of individuals from diverse geographical locations infected with KSHV showed that gB was highly conserved [30] and that KSHV infectivity could be neutralised by rabbit anti-gB antibodies [29]. Besides gB, the gH-gL heterodimer is required for fusion [31], and is hypothesized to play an important role specifically in the post-binding steps of KSHV infection, as treatment with anti-gH and anti-gL antibodies inhibited KSHV infection of target cells without blocking binding of the virus to the cell surface [20]. Recently, gH-gL has also been found to bind to KSHV entry receptors on host cells [32].

The KSHV K8.1 gene is alternatively spliced to form two separate glycoproteins, K8.1A and K8.1B, with the A protein being dominant on the viral envelope [22,33,34]. Like gB, K8.1A also facilitates attachment to target cells by binding to heparan [33], but it is not necessary for infection [35].

## 3. Host Cellular Binding and Entry Receptors Used by KSHV

Like several other viruses, KSHV engages two kinds of receptors to enter cells: binding and entry receptors. The first step in KSHV infection of target cells is binding of the virus to the cell membrane, which is followed by transfer to the actual entry receptor or receptor complex. These receptors are dependent on the target cell type. KSHV’s broad cell tropism is owed to the number of different entry receptors it can potentially utilize.

### 3.1. KSHV Cellular Binding Receptors

On most cells, binding of KSHV is facilitated by interaction of the gB and K8.1A viral glycoproteins with ubiquitous HSPGs [29,33,36,37]. There is also evidence that the KSHV complement control protein (KCP), encoded by ORF4, binds to HSPGs [38]. HSPGs are well known to be used by different viruses as an attachment platform to target cells, owing to their abundant expression on cell surfaces [11]. Binding to HSPGs is thought to concentrate the virus on the cell surface to enhance infection. This is supported by binding studies using soluble forms of gB and K8.1A, made in baculovirus. The binding of these proteins to target cells could be blocked by soluble heparin [33,39], and gB and K8.1A could also be eluted by high concentrations of soluble heparin, but not chondroitin sulphate, a related glycosaminoglycan [29,33]. In infection studies of susceptible human dermal microvascular endothelial cells (HMVEC-d) pre-treated with soluble heparin, KSHV infection was blocked in a dose-dependent manner, however pre-treatment with chondroitin sulphate did not prevent infection [35], further demonstrating the specific binding of KSHV to HSPGs.

Moreover, it was shown that low or defective expression of Ext1, the enzyme responsible for the glycosylation of heparan sulphate (HS) [40], resulted in reduced expression of HS which consequently resulted in reduced KSHV infection. In fact, expression of Ext1 was found to be very low in several B cells and B cell lines, which are known to be refractory to KSHV infection [41]. Overexpression of Ext1 in the murine B cell lines A20 and M12 was associated with enhanced infection by Murine Gamma Herpesvirus 68, a murine herpesvirus phylogenetically related to KSHV [42,43]. Furthermore, the expression of HS on BJAB cells was able to promote KSHV binding to the cell surface, but not infection [41].

These studies demonstrate the importance of HS in facilitating binding of KSHV to target cells with consequences for downstream infection, although not strictly essential for entry on all cell types [44].

### 3.2. KSHV Cellular Entry Receptors

The following paragraphs briefly introduce the various receptors KSHV has been found to engage, while a more detailed description of these receptors depending on the cell type is found in Section 4 and summarized in Table 1.

#### 3.2.1. DC-SIGN

Dendritic cell specific intercellular adhesion molecule-3 (ICAM-3) grabbing non-integrin (DC-SIGN; CD209) is a C-type lectin present on antigen presenting cells. It has numerous functions including adhesion, migration, and signaling [58]. DC-SIGN has been identified as a receptor for several other viruses, including HIV-1 and bunyaviruses [59,60]. It has been identified as a receptor for KSHV on several cell types, including B cells, macrophages, and dendritic cells. Transfection of this receptor into KSHV-unsusceptible cells can render them permissive to infection [46,48,49,50].

#### 3.2.2. Eph Receptors

Eph receptors are a family of receptor tyrosine kinase molecules expressed on cell surfaces. They play a role in development, pathway finding by axons and vascular formation [61,62,63], but some of them have also been identified as cellular entry receptors for KSHV and play a particularly important role in the infection of endothelial cells [51]. There are 14 different Eph receptors, divided into EphA and EphB classes by the homology of their extracellular domains. Ephrins A2, A4, A5 and A7 have been implicated in KSHV infection. Under normal physiological conditions EphA2 is known to play a key role in several developmental processes, cell migration, adhesion and trafficking, while EphA4, EphA5 and EphA7 are largely involved in the development of the nervous system [63].

#### 3.2.3. Integrins

Integrins are a large family of extracellular cell surface receptors, largely responsible for extracellular matrix (ECM) interactions with cells and outside-in signaling events [64]. They function as transmembrane linkers between the ECM and the actin cytoskeleton of animal cells, and often cooperate with other receptors to promote cell growth, cell survival, and cell proliferation. For example, α3β1 plays an important role in establishing and maintaining epithelial tissues [65], while the αV integrins such as αVβ3 and αVβ5 bind a broad range of cell adhesion proteins such as vibronectin and play a role in angiogenesis [66,67]. Integrin α5β1, also known as the fibronectin receptor, has functions in angiogenesis, particularly in endothelial cells during development. It may also act as a regulator of angiogenic signals [68].

Several viruses engage more than one integrin for infecting target cells and signaling to enhance internalization [69]. Integrins have been shown to play a critical role in KSHV infection [45,57], and indeed, KSHV gB was found to possess an integrin binding Arginine-Glycine-Aspartate (RGD) motif, potentially interacting with the integrins α3β1, αVβ3 and αVβ5, and α5β1 [45].

#### 3.2.4. xCT

xCT is the 12-transmembrane glutamate/cysteine exchange transporter [69]. It is part of a cell surface 125-kDa disulphide linked heterodimeric membrane protein CD98, a larger complex containing a glycosylated heavy chain and a group of light chains, of which xCT makes up one. The CD98 molecule plays roles in cell adhesion, integrin activation and virus induced cell fusion [70,71]. xCT was identified to play a role in KSHV infection in multiple susceptible cell lines including Vero cells, human PCI-13 (head-and-neck squamous cell carcinoma), and Mel-1700 (melanoma) cells. Moreover, xCT transfection into non-permissive cell lines rendered them susceptible to KSHV infection [72].

## 4. Cell-Type Specific Expression of Surface Receptors Used by KSHV for Cellular Entry

### 4.1. Endothelial Cells and Fibroblasts

Infection of endothelial cells is central in the development of KS, the most common KSHV-associated pathology, as KS tumors are thought to be of endothelial cell origin [73]. Endothelial cells have been shown to express several receptors utilized by KSHV. Although the predominant cell type in KSHV tumors are typically endothelial cells, or KSHV-infected “spindle cells”, fibroblasts are also found in KS lesions and can be infected by KSHV [74].

#### 4.1.1. Integrins and xCT as KSHV Receptors on Endothelial Cells and Fibroblasts

As mentioned previously, the KSHV glycoprotein gB contains the integrin binding RGD sequence [19,45] which is known to facilitate interactions between several extracellular matrix proteins and host cell integrins [75]. Peptides directed towards RGD sequences significantly inhibited infectivity of KSHV in human foreskin fibroblast (HFF) cells, indicating for the first time a role for integrins in herpesvirus infection of target cells [57]. Endothelial cells and fibroblasts express the α3β1 integrin at high levels in vivo [76] and anti-α3β1 antibodies were found to partially inhibit infection of HMVEC-d cells by GFP-conjugated KSHV [57]. Interestingly, soluble α3β1 inhibited virus internalization but not binding to target cells; therefore, this integrin is likely involved in the cell entry phase of infection [57]. Importantly, the incomplete inhibition of KSHV internalization by targeting α3β1 might be due to KSHV recognizing multiple receptors on endothelial cells and fibroblasts and utilizing them for entry along with α3β1. It was found that αVβ3 and αVβ5 were also involved in KSHV infection, and, on HMVEC-d cells, act as a multimolecular complex with α3β1 and xCT [45]. When treated with soluble forms of these three integrins individually, KSHV infection of HMVEC-d cells and HFF cells was blocked between 35–60%. Furthermore, it was determined that xCT associates with this integrin complex in HMVEC-d cells, and while antibodies against xCT did not block viral binding or viral entry into the cells, KSHV gene expression was inhibited in the nucleus, suggesting xCT plays a role in the post entry stage of KSHV infection [45]. Further studies on the formation of lipid rafts, an essential process for the endocytosis of KSHV into endothelial cells, suggest that xCT plays an essential role in signaling for entry [77]. However, it still needs to be determined which KSHV glycoprotein interacts with xCT to conclusively elucidate its function as an entry receptor.

#### 4.1.2. EPHA2 as a KSHV Receptor on Endothelial Cells and Fibroblasts

The Eph receptor A2 protein (EPHA2) was demonstrated to be a master assembly regulator for KSHV entry and signal molecules in endothelial cells [51,52,53]. Hahn et al. showed that EPHA2 interacts directly with KSHV gH-gL which resulted in EPHA2 phosphorylation, and knockout of both alleles for this receptor in a murine model blocked KSHV infection of endothelial cells. Overexpression of EPHA2 resulted in increased KSHV infection, proportional to the expression level [51]. In agreement, a separate study using HMVEC-d cells found that pre-treatment of KSHV with soluble EPHA2, using short hairpin RNA (shRNA) against EPHA2 or treating the endothelial cells with monoclonal antibodies towards EPHA2 significantly reduced KSHV internalization and viral gene expression [52].

A study aiming to define key molecules in the regulation of KSHV entry to HFF cells established that KSHV stimulates the association of integrins with EPHA2 [53]. Knockdown of EPHA2 in HFF cells resulted in significant blocking of KSHV infection, measured by determining internalized viral DNA, showing that EPHA2 is also a receptor for KSHV in fibroblasts [53].

A recent high-resolution structural investigation into the complex formation between KSHV gH-gL and the ligand binding domain (LBD) of EPHA2 revealed primarily gL protein binding to LBD [78]. It was further revealed that many amino acids of EPHA2′s LBD are potentially recognized by other γ-herpesviruses, thereby providing the structural basis of EPHA2 recognition by γ-herpesvirus gHgL [78]. Recently, an investigation of EPHA2 sequence variants in the protein coding region of the gene in South African HIV positive individuals revealed that certain variants, primarily located in the functionally important tyrosine kinase domain, affected patient’s susceptibility to KSHV infection or KS development [79]. This further supports the current understanding that EPHA2 plays an important role in KSHV entry and may be involved in signaling events leading to KS development.

However, it remains to be understood how this receptor mechanistically contributes to pathogenesis of KSHV infection and/or tumorigenesis following KSHV infection. Additionally, incomplete reduction of infection upon EPHA2 knock out in endothelial cells and fibroblasts suggests that additional receptors are used by KSHV for entry, likely other EPH receptors. While EPHA2 has garnered interest as a target for therapeutic intervention, the role of other receptors in KSHV entry would need to be teased apart before such a development.

### 4.2. B Cells, Macrophages, Dendritic Cells and Monocytes

B cells are considered the main latent reservoir for KSHV in vivo and are the primary cell type involved in PEL and MCD [80,81]. This is perplexing because B cells are notoriously difficult to infect with KSHV in vitro [41,81,82], and for a long time little was known about how this reservoir develops. Lack of expression of appropriate amounts of HS and the differential expression of appropriate viral entry receptors have been suggested to explain why B cells and B cell lines are not permissive to KSHV infection in vitro [41,49]. Moreover, other immune cells such as macrophages, dendritic cells and monocytes may be infected with KSHV and play an important role in the inflammatory microenvironment of KSHV-associated pathologies.

#### 4.2.1. DC-SIGN as a KSHV Receptor on B Cells, Macrophages and Dendritic Cells

Previous findings from Rappocciolo et al. implicate DC-SIGN as a KSHV receptor for dendritic cells and macrophages [50]. DC-SIGN, a C-type lectin receptor primarily involved in innate immune recognition, has been found to bind KSHV gB through its carbohydrate recognition domain (CRD) [48]. Indeed, infection of dendritic cells and IL-13 treated macrophages could be blocked by pre-treatment with an anti-DC-SIGN monoclonal antibody, and cell lines not normally susceptible to KSHV infection, such as K562 and B-LCL cells, were permissive to KSHV infection when transfected to overexpress DC-SIGN [50]. Therefore, it was hypothesized that DC-SIGN may be an entry receptor for KSHV on B cells since DC-SIGN was found to be expressed on activated B cells at a higher level than non-activated B cells. Radiolabelled KSHV binding to differentially activated B cells supported this observation as B cells activated with CD40 and IL-20 displayed much higher virus binding than non-activated B cells. However, low levels of KSHV binding to non-activated cells were attributed to low levels of DC-SIGN or HS. Pre-treatment of the activated B cells with either a monoclonal antibody against DC-SIGN or mannan, a natural ligand for DC-SIGN, inhibited infection in a dose-dependent manner [49].

#### 4.2.2. Eph Receptors as KSHV Receptors on B Cells

A system to infect B cells with KSHV in vitro has been developed by co-culturing SLK endothelial cells infected with recombinant KSHV (rKSHV) conjugated to GFP with a number of lymphoid cell lines including uninfected BJAB cells and cell lines derived from (KSHV-positive) PEL patients. All cell lines usually resistant to cell-free infection with KSHV were found to be GFP positive, including the PEL cell lines which already harboured a KSHV genome, showing that they can be superinfected [83]. Employing this method of infection, Hahn et al. demonstrated that soluble forms of the Eph family receptors were able to block cell-to-cell transmission of KSHV [55]. To determine precisely which Eph receptors were responsible for facilitating KSHV entry into BJAB cells, soluble KSHV gH-gL proteins were used to determine their binding partners in BJAB cell lysates. Pulldown experiments revealed that EPHA7 and, to a lesser extent, EPHA5 were bound to the gH-gL complexes [56]. Knockout of these two receptors on BJAB cells resulted in an 84% and 57% reduction of KSHV infection, respectively [56]. Additionally, EPHA7 was found to be expressed at high levels in a PEL cell lines, along with EPHA2, suggesting a role for these two receptors in the infection of cells that eventually develop into PEL [56]. Using the human lymphoma B cell line MC116, a role for gH in infection could not be conclusively established; it was therefore suggested that KSHV entry into B cells is mediated through the K8.1 envelope glycoprotein (see also Section 4.2.4.). Indeed, MC116 cells were shown to express EPHA4 and EPHA7 which have been implicated in KSHV infection in both cell-free and cell-to-cell settings [54,56]. Again, the role of EPH receptors in KSHV infection of B cells and subsequent pathogenesis remains only partially understood and it remains to be seen if the abundance of EPH expression on primary B cells correlates with permissiveness to KSHV infection and PEL development.

#### 4.2.3. Integrins and DC-SIGN as KSHV Receptors on Monocytes

Monocytes are key in the inflammatory pathogenic response in KS, suggesting their ability to be infected with KSHV. Using the monocytic cell line THP-1, Kerur et al. suggested that after initial binding to HSPGs, DC-SIGN acted as an entry receptor for KSHV. Indeed, when cells were pre-incubated with a monoclonal antibody against DC-SIGN, viral internalization was reduced by 43% [46]. Furthermore, pre-incubation of THP-1 cells with soluble forms of the integrins α3β1, αVβ3, αVβ5 and α5β1 resulted in various levels of reducing viral gene expression, with α3β1 blocking 94% of ORF73 expression. Confocal microscopy further revealed co-localization of the virus with these integrins, demonstrating their important role as KSHV entry receptors on monocytes [46].

#### 4.2.4. Unidentified KSHV Receptors on B Cells

Recently, an essential role for the K8.1 glycoprotein in KSHV infection of MC116 cells was identified [44]. Using anti-K8.1A antibodies, KSHV infection of these cells (in contrast to Vero and HEK cells) was inhibited. Moreover, tonsillar B cells could also be inhibited between 60–80% by pre-treatment with K8.1A monoclonal antibodies. Surprisingly, this was independent of HSPG binding, suggesting that there are other KSHV receptors interacting with K8.1A that are yet to be discovered and would shed more light on the understanding of how KSHV infects B cells [44].

### 4.3. Epithelial Cells

As KSHV is thought to be mainly transmitted through saliva [84], oral epithelial cells may play a key role in KSHV transmission. However, little is known about in vivo infection of epithelial cells in this environment, representing an important arena for future study, particularly relevant for the development of prophylactic interventions to initial KSHV infection.

#### 4.3.1. Integrins as KSHV Receptors on Epithelial Cells

Pre-incubation of Vero cells with soluble forms of the integrins α3β1, αVβ3 and αVβ5 before KSHV infection displayed variable but significant inhibition, and upon treatment with a combination of integrins cumulative inhibitory effects were observed [45]. A study by Garrigues et al. identified a human salivary gland (HSG) epithelial cell line (recently identified to actually have been HeLa cells after some controversy [85]) which lacked integrin αVβ3 but expressed high levels of other putative KSHV binding and entry receptors, including HS and α3β1. Despite normal expression of these receptors, the cell line was refractory to both KSHV infection and binding [85]. However, re-introducing αVβ3 integrin into the cells allowed KSHV to bind and infect them, determined by fluorescently visualising the co-localization of KSHV and α3β1 on the cell surface [85]. While this is an interesting result, the study lacked strong quantitative analysis as it was mainly reliant on immunofluorescent imaging.

#### 4.3.2. Eph Receptors as KSHV Receptors on Epithelial Cells

Hahn et al. implicated EPHA2 as a KSHV receptor for both endothelial (see Section 4.1.2.) and epithelial cells [51]. Indeed, pre-treatment of cells with soluble EPHA2 inhibited KSHV infection up to 90% in all cell lines observed, and a similar reduction of infection was seen when pre-treating cells with the soluble EPHA2 ligand, ephrinA4 [51].

KSHV is thought to engage both EPHA2 and integrins for entry into target cells, but a recent gene editing approach demonstrated KSHV infection of the epithelial cell lines Caski-1 and HeLa to be independent of the integrins α3β1, αVβ3, and αVβ5 [47]. Knocking out these integrins by CRISPR/Cas9 did not lead to reduced infectivity which contrasts the study by Garrigues et al. [85]. Knockout of EPHA2, however, resulted in a significantly reduced but not completely abolished level of infection. It was further determined that multiple Eph receptors, including EPHA4 and EPHA5, could rescue KSHV infection, and interestingly, EPHA4 overexpression greatly enhanced infection, suggesting its important role in epithelial cell infection [47]. This was confirmed by Chen et al. demonstrating that epithelial cell overexpression of EPHA4 promotes KSHV fusion activity to a greater extent than EPHA2 [54]. When both EPHA2 and EPHA4 were knocked out in HEK293T cells, fusion activity dramatically decreased compared to single knockouts, and reintroduction of either receptor partially restored function while reconstitution of both restored complete function. This study also showed that both EPHA2 and EPHA4 bind to KSHV gH-gL and mediate fusion with this viral glycoprotein complex [54], further confirming that both EPHA2 and EPHA4 play an important role in KSHV infection of epithelial cells.

## 5. Conclusions

While several advances have recently been made in the quest to elucidate the early steps of KSHV engagement with its host cell, a better understanding of the entry process, particularly in a cell-type specific context, may aid in preventing KSHV-associated pathologies. This review summarized the known host–virus interactions that have been identified in recent years with focus on the broad host tropism of KSHV.

The importance of Eph receptors in KSHV infection, particularly of endothelial cells, is becoming increasingly apparent and will be essential in preventing, diagnosing, and treating KS, the most common KSHV-associated pathology, and other KSHV-associated diseases. Moreover, the identification of a cellular binding partner for the viral glycoprotein K8.1 will bring us closer to understanding infection of B cells, one of the most important target cells in several other KSHV-associated pathologies. Finally, elucidating the mechanism of epithelial cell infection, assumed to be one of the initial cell types that come into contact with KSHV via saliva, could result in novel preventative means against early KSHV infection.

Importantly, while in vitro studies of KSHV infection are necessary in gaining insight into general viral entry mechanisms, studies involving patient cohorts will identify the clinical relevance of the molecular host–virus interactions which is essential in the development of novel diagnostic approaches.

## Figures and Tables

**Table 1:** Summary of the known KSHV receptors on susceptible cell types.

Receptor	Cell Type
Binding/Attachment	
Heparan sulphate proteoglycans (HSPGs)	Endothelial cells, fibroblasts [45] Monocytes [46] Epithelial cells [47]
DC-SIGN	B cells, macrophages and dendritic cells [48,49,50]
**Entry**	
DC-SIGN	B cells [49] Macrophages, dendritic cells, monocytes [50]
EPHA2	Endothelial cells [51,52] Fibroblasts [53] Epithelial cells [51]
EPHA4	Epithelial Cells [47,54]
EPHA5	B cells [55,56]
EPHA7	B cells [55,56]
α3β1	Endothelial cells [45,57] Fibroblasts [45,57] Epithelial cells [45]
αVβ3	Endothelial cells [45] Fibroblasts [45]
αVβ5	Endothelial cells [45,57] Fibroblasts [45,57] Epithelial cells [45] Monocytes [46]
α5β1	Monocytes [46]
xCT	Fibroblasts [45] Endothelial cells [45]

## Data Availability

The data that support the findings of this article are openly available at PubMed (https://pubmed.ncbi.nlm.nih.gov/).
